# Stem Cell-Like Dog Placenta Cells Afford Neuroprotection against Ischemic Stroke Model via Heat Shock Protein Upregulation

**DOI:** 10.1371/journal.pone.0076329

**Published:** 2013-09-25

**Authors:** SeongJin Yu, Naoki Tajiri, Nick Franzese, Max Franzblau, EunKyung Bae, Simon Platt, Yuji Kaneko, Cesar V. Borlongan

**Affiliations:** 1 Department of Neurosurgery and Brain Repair, University of South Florida College of Medicine, Tampa, Florida, United States of America; 2 Department of Small Animal Medicine & Surgery, College of Veterinary Medicine, University of Georgia, Atlanta, Georgia, United States of America; University of South Florida College of Medicine, United States of America

## Abstract

In this study, we investigated the dog placenta as a viable source of stem cells for stroke therapy. Immunocytochemical evaluation of phenotypic markers of dog placenta cells (DPCs) cultured in proliferation and differentiation medium revealed that DPCs expressed both stem cell and neural cell markers, respectively. Co-culture with DPCs afforded neuroprotection of rat primary neural cells in a dose-dependent manner against oxygen-glucose deprivation. Subsequent in vivo experiments showed that transplantation of DPCs, in particular intravenous and intracerebral cell delivery, produced significant behavioral recovery and reduced histological deficits in ischemic stroke animals compared to those that received intra-arterial delivery of DPCs or control stroke animals. Furthermore, both in vitro and in vivo studies implicated elevated expression of heat shock protein 27 (Hsp27) as a potential mechanism of action underlying the observed therapeutic benefits of DPCs in stroke. This study supports the use of stem cells for stroke therapy and implicates a key role of Hsp27 signaling pathway in neuroprotection.

## Introduction

Stroke is a major cause of death and impairment in both humans and dogs. Stroke is a constantly expanding area of research, but there exists a paucity of studies investigating stroke in dogs. Currently, there exists no specific treatment for ischemic stroke in dogs. While the clinical outcome of dog ischemic stroke is considered fair to good, surviving animals demonstrate significant risk for the development of new acute neurological symptoms and death [1], thereby warranting novel treatment interventions.

Stem cell therapy has been evaluated for a variety of different diseases, including neurological disorders such as stroke [2], [3], [4], [5]. Stem cells range in cell potency and with the correct signals, give rise to many different cell types that make up the organism. In addition to embryonic stem cells, tissue-specific stem cells can be isolated at more advanced developmental stages, such as hematopoietic stem cells [6], [7]. Due to recent claims that they exhibit remarkable plasticity in development when placed in new environments, adult stem cells are an attractive source of cells for therapy [8]. Accumulating evidence supports the therapeutic potential of mesenchymal stem cells (MSCs) as transplantable donor cells due to their ability to self-renew, proliferate, and differentiate into a variety of cell types. The placenta not only contains hematopoietic precursors [9], but also cells exhibiting characteristics of bone marrow-derived MSCs with a high degree of plasticity [10]. Further studies have indicated that these cells seem to be an acceptable alternative source for human MSCs [11], which have also been isolated from in-third-trimester placenta, amnion, amniotic fluid, chorion, Wharton jelly, and umbilical cord vein walls [12], [13], [14], [15], [16], [17], [18]. Recently, stem cell transplantation has been shown to be safe and effective for treating stroke in pre-clinical studies. For example, transplanted MSCs from the human placenta demonstrated reduced stroke deficits in rats [19], [20], [21]. Because the envisioned product is autologous placenta cell transplant in dogs, we embarked in this study to characterize the efficacy of dog placenta-derived MSCs (DPCs) in a rodent stroke model. Along this line, the results from this study may provide insight to autologous placenta cell transplantation in humans.

Promising preclinical results from stem cell therapy in stroke models has provided the impetus to enter clinical trials [22], [23], [24], [25], while the mechanism of action is not fully understood. We hypothesize that stem cells possess therapeutic proteins which aid in ameliorating the damaged neuronal micro-environment architecture that is associated in stroke. To this end, we examined heat shock proteins (Hsp), which are highly conserved and act as a molecular chaperone and/or have anti-apoptotic activities [26]. The expression of Hsp27 in the brain is notable because this protein is highly inducible in glial cells and neurons following a wide range of noxious stimuli including ischemia, epileptic seizure, and hyperthermia [26], [27], [28]. Interestingly, alterations in glial and neuronal survival accompany stroke [29]. It is highly contested whether glial cells or neurons are more easily damaged by stroke; however, the improved survival of neurons has been linked to the survival of glial cells [30]. Following an ischemic injury, glial cells undergo gliosis characterized by hypertrophy, upregulation of nestin intermediate filaments, and activate cell proliferation [31]. A diminished immunoreactivity of glial and neuronal markers has been found to be an early and sensitive marker of ischemic damage after permanent and transient focal stroke [32], [33]. Hsp27 has been characterized as a stress protein known to be expressed differently after focal ischemia with regard to cell type, regional distribution, and injury-reperfusion times [34], [35]. Hsp27 is reported to be widely inducible in both glial and neuronal cells of peri-lesional and remote areas after injury [36], [37]. Hsp27 expression may be a potent therapeutic pathway for the neuroprotection afforded by stem cell therapy. Rats subjected to a middle cerebral artery occlusion (MCAo) and treated with neural stem cell transplant exhibited upregulation of Hsp27, resulting in neuroprotection against apoptosis [38]. Furthermore, rats subjected to ischemic stroke and treated with human umbilical cord MSCs (hUCMSCs) showed an upregulated expression of Hsp27 while demonstrating a significant improvement in neurological deficit in comparison to those treated with hUCMSCs without upregulated expression of Hsp27 [39]. Accordingly, we examined the role of Hsp27 and the neuroprotective effects produced by transplanted DPCs in experimental stroke. In this study, we showed that the transplantation of DPCs afforded therapeutic benefits via upregulation of Hsp 27 in both in vitro and in vivo models of stroke.

## Materials and Methods

### Characterization of DPCs in culture

Isolation of cells from discarded dog placenta was processed within 24 hours after normal deliveries under University of Georgia IACUC guidelines. Dog owners signed informed consent forms to use the placenta for the present study. No live dogs were utilized in this study. The umbilical vein was flushed with 10 ml of low-glucose type DMEM (DMEM-LG) (Gibco). The vein was filled with 0.5% crude collagenase in phosphate-buffered saline (PBS) and both proximal and distal ends were clamped. After incubation at 37°C for 15 min, the collagenase solution was collected and centrifuged for 5 min at 300× g. Cell pellets were re-suspended in DMEM-LG containing 20% fetal bovine serum, 2 mM L-glutamine, 100 U/ml penicillin G, 100 µg/ml streptomycin and 10 ng/ml. Cells were seeded in 25 cm^2^ culture flasks at the concentration of 2×10^4^ cells/cm^2^. Cultures were maintained at 37°C in a humidified atmosphere containing 5% CO_2_. After culture for 3 days non-adherent cells were removed and thereafter the medium was changed every 3 days. Well-developed colonies of fibroblast-like cells appeared one week later. Once cell confluence was achieved in the culture flasks, the cells were detached from the bottom using Hank's balanced salt solution containing 0.125% trypsin and 1 mM EDTA. After several washings, 2×10^4^ cells/cm^2^ were seeded into 75 cm^2^ culture flask for the subculture.

### Immunocytochemistry

Subcultured DPCs were washed with PBS and then fixed with 4% paraformaldehyde (PFA) for 30 mins at room temperature. Following several washings, cells were permeabilized with 1% Triton X-100 for 20 min. After incubation in 20% normal goat serum in PBS for 1 hr, cells were reacted overnight at 4°C in the presence of one of the following antibodies against the antigens: Oct-4 (1∶100, Abcam), Nanog (1∶200, Abcam), Nestin (1∶50, Abcam), SSEA (1∶500, Abcam), CXCR4 (1∶100, Abcam), MAP2 (1∶1000, Chemicon), NeuN (1∶100, Abcam), GFAP (1∶500, Sigma) and O4 (1∶100, Abcam). Washed cells were incubated with goat anti-rabbit IgG (Alexa Fluor 488, Invitrogen) or goat anti-mouse IgG (Alexa Fluor 488, Invitrogen) for 90 min at room temperature. Samples were washed five times to remove unbound antibody. For visualization, each slide was covered with Crystal Mount (Sigma). A Zeiss Imager fluorescence microscope was used to evaluate the cells.

### Evaluation of neuroprotection by DPCs when co-cultured with primary rat cells in OGD condition

Mixed rat primary astrocytes-neurons (1∶1 ratio) (embryos at Day 18; BrainBits) (1×10^5^ cells/well) seeded onto the upper chamber of a Boyden chamber (Costar Transwell assay, Corning, NY, USA) supplemented with NbActive4 (BrainBits) in the absence of antibiotics. The chamber containing the rat primary neural cells was placed in a 24-well plate containing subcultured DPCs cells using different co-culture ratio of DPCs to rat primary neural cells as follows: 1∶1 (100%), 1∶2 (50%), 1∶4 (25%), and 1∶0 (0%). This co-culture system was subsequently exposed to the OGD injury model as described previously [18] with a few modifications. Briefly, the culture medium was replaced by a glucose-free Earle's balanced salt solution with the following composition: 116 mM NaCl, 5.4 mM KCl, 0.8 mM MgSO_4_, 1 mM NaH_2_PO_4_, 26.2 mM NaHCO_3_, 0.01 mM glycine, 1.8 mM CaCl_2_, and pH adjusted to 7.4. The co-culture system was placed in a humidified chamber and equilibrated with continuous flow of 92% N_2_ and 8% O_2_ gas for 15 minutes. After this equilibrium, the upper chamber containing only the rat primary neural cells was sealed and placed into the incubator at 37°C for 4 hours and 12 hours for MTT assay and Trypan blue stain, respectively [18].

### Cell viability

Cell viability was evaluated by ATP activity following the supplier's protocol (Promega, WI) and by Trypan blue (Sigma, MO). MTT assay was conducted by adding MTT assay solution immediately after OGD. The intensities of chemiluminescence of ATP activity were measured and calculated by Image station 2000R system (Kodak, NY). In addition, Trypan blue exclusion method was conducted and mean viable cell counts were calculated in three randomly selected areas (0.2 mm^2^) in each well (n = 5 per treatment condition) to reveal the cell viability for each treatment condition.

### Transplantation of DPCs in an in vivo stroke model

Adult Sprague-Dawley male rats, weighing between 220 g and 250 g and around 2 months old, served as subjects in the present study. The animals were housed in a temperature-controlled room with normal 12–12 h light-dark cycle. Food and water were freely available in the house cage. All animal (rat) work, including necessary precautions to minimize any stress related to surgical procedures and behavioral testing of animals in this study, was conducted according to approved IACUC guidelines of the University of South Florida. Animals were anesthetized with gas inhalation composed of 30% oxygen (0.3 L/min) and 70% nitrous oxide (0.7 L/min) mixture. The gas was passed through an isoflurane vaporizer set to deliver 3% to 4% isoflurane during initial induction and 1.5% to 2% during surgery. Transient unilateral focal ischemia was produced using a well-established middle cerebral artery occlusion (MCAo) using the intraluminal suture model as previously described [19]. The body temperature of the animals was maintained at 37°C during the surgery until they recovered from anesthesia. The fluorescent tracker dye, PKH26 was applied to DPCs to track cells in the rat brain. Briefly, cells that were cultured in 10% FBS/DMEM for 4 days were re-suspended in 1 ml diluent solution (Sigma, USA) and mixed for 2 min with 1 ml of 2 µM PKH26 (Sigma, USA). The labeled cells were visualized using a rhodamine filter. The PKH26 dye is suitable for labeling cell populations to track the long-term cell migration in vivo, since it does not physically weaken the cell membrane and is not transferred to other cells. Cells were transplanted intravenously (IV, 1 million viable DPCs) via the jugular vein, intra-arterially (IA, 1 million viable DPCs) via the internal carotid artery, or intracerebrally (IC, 200,000 viable DPCs) via stereotaxic guided approach targeting the striatum and cortex, at 3 hours post-MCAo. A separate cohort of stroke animals served as control group, and received vehicle only. Subject size was 10 per group, except in the control group which was 12 (4 rats per cell delivery route). DPC viability, assessed from leftover cells in microvials, at pre- and post-transplantation periods were >95% and >80%, respectively.

### Behavioral analysis

Assessment of stroke deficits was based on motor asymmetry (elevated body swing test, EBST) and neurological function (Bederson test) at 72 hours after stroke just prior to euthanasia as previously described [40], [41], [42]. The analyses of both EBST and Bederson data utilized raw individual scores [19]. To prevent any examiner's bias, all behavioral evaluations were performed by an investigator blinded to the treatment conditions.

### Immunohistochemistry

Under deep anesthesia, rats were sacrificed at 1 week after reperfusion, and perfused through the ascending aorta with 200 ml of cold PBS, followed by 200 ml of fresh 4% PFA in PBS. Brains were removed and post-fixed in the same fixative for 3 days followed by 30% sucrose in phosphate buffer for 1 week. Six series of coronal sections were cut at a thickness of 30 µm by cryostat and stored at −20°C until processing. Free floating sections for immunohistochemistry were incubated overnight at 4°C with an anti-NeuN antibody (1∶50, Abcam), anti-MAP2 (1∶100, Chemicon), anti-GFAP (1∶500, Chemicon), and anti-Hsp27 antibody (1∶100, Abcam) with 10% normal goat serum. Finally, brain sections were counterstained with Nissl stain. Immunofluorescent and light microscopy was carried out using Zeiss imager.

## Results

### Phenotypic characterization of cultured DPCs

**Figure 1:**
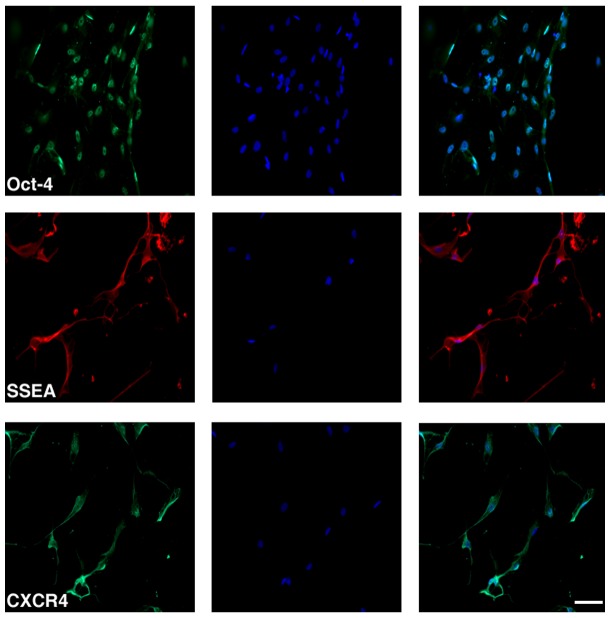
DPCs display stem cell surface markers. Subcultured DPCs at day 3 in culture expressed stem cell phenotypes including Oct-4, SSEA, and CXCR4. Left panels: phenotypic marker; Middle panels: DAPI; Right panels: Merged. Scale bar  = 75 µm.

**Figure 2:**
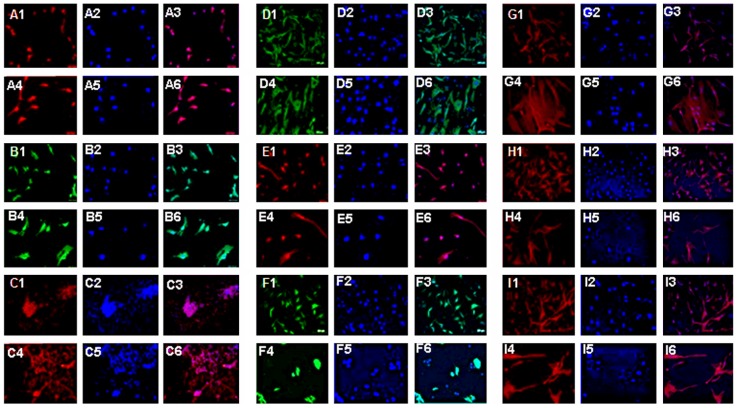
DPCs maintain stemness and differentiate into neural cells under appropriate conditions over multiple passages. Stem cell under proliferation medium and neural cell markers under differentiation medium were detected at passage 10. The DPCs expressed the stem cell markers, Oct-4 (A), SSEA (B), Nanog (D), and CXCR4 (E), and the neural cell markers, NeuN (C), GFAP (F), Nestin (G), MAP2 (H), and O4 (I). 1,4: phenotypic marker; 2,5: DAPI; 3,6: Merged. 1–3: 10X; 4–6: 20X.

Under proliferation and differentiation media, DPCs expressed stem cell (Figure 1) and neural cell markers, respectively, at passage 10 (Figure 2) and even up to passage 20 (data not shown). The DPCs expressed the stem cell markers, Oct-4 (92%–96%), Nanog (94%–97%), SSEA (88%–94%), and CXCR4 (91%98%), and the neural cell markers, Nestin (55%–64%), MAP2 (25%–32%), NeuN (18%–26%), GFAP (42%–56%), and O4 (12%–17%).

### Co-culture with DPCs protects rat neural cells from OGD

**Figure 3:**
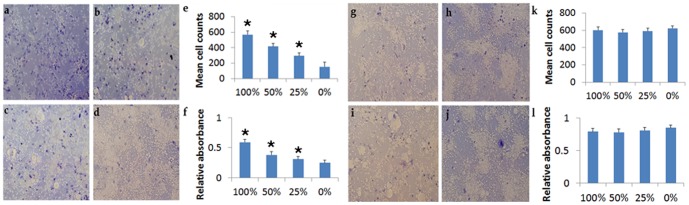
DPC co-culture protects rat neural cells against OGD. Co-culture of rat primary neural cells with DPCs afforded neuroprotection against OGD dose-dependently in both Trypan blue and MTT activity assays of cell viability (a–f). The percentages correspond to the ratio of DPCs co-cultured with rat primary neural cells as follows: 1∶1 (100%), 1∶2 (50%), 1∶4 (25%), and 1∶0 (0%). DPCs co-cultured with rat primary neural cells under non-OGD condition did not alter cell viability in both assays (g–l). Asterisk* corresponds to statistically significant difference (p<0.05 vs. 0%). a,g: 0%; b,h: 25%; c,i: 50%; d,j: 100%; e,k: Trypan blue stain quantification; f,l: MTT assay quantification.

Co-culture of rat primary neural cells with DPCs afforded neuroprotection against OGD, as assessed by Trypan blue and MTT assay (Figure 3). Cell viability of rat primary neural cells with DPCs exhibited a dose-dependent response, with high doses (100% and 50% corresponding to a 1∶1 ratio and 1∶2 ratio of rat cells and DPCs in co-culture, respectively) demonstrating significantly much improved cell survival and ATP activity versus the low 25% dose (1∶4 ratio of rat cells and DPCs in co-culture) and the vehicle (0%). ANOVA revealed significant treatment effects (F_3,9_ = 43.18, p<0.001) and pairwise comparisons between doses were significant at p<0.05. Co-culture of rat primary neural cells with DPCs under non-OGD condition did not alter cell viability in both assays.

### Dose-dependent DPC modulation of GFAP, NeuN, and MAP2 expression in OGD-exposed primary rat cells

**Figure 4:**
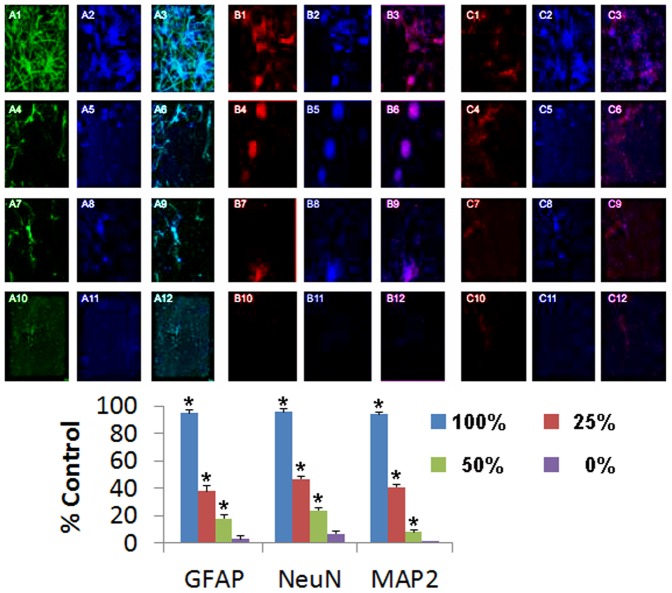
DPCs dose-dependently facilitate GFAP, NeuN, and MAP2 expression in primary rat cells post-OGD. Immunocytochemical analyses revealed that co-culture of DPCs modulated dose-dependent expression of GFAP, NeuN, and MAP2 in OGD-exposed primary neural cells in the following order: 100%>50%>25%>0%. Phenotypic markers labeled as A: GFAP; B: NeuN; C: MAP2; Immunofluorescence identified as 1: phenotypic marker; 2: DAPI; 3: Merged; Cell doses given as 1–3: 100%; 4–6: 50%; 7–9: 25%; 10–12: 0%. Quantitative analyses revealed dose-dependent expression of these neural markers (*p<0.05 vs. controls).

Expression of GFAP, NeuN, and MAP2 was dose-dependently modulated by DPC co-culture following OGD (Figure 4). The high doses of 100% and 50% exhibited increased neural phenotypic expression compared to 25% DPCs or standard medium without DPCs (0%). Quantitative analyses revealed significant dose-dependent expression of these neural markers (Figure 4). ANOVA for each phenotypic marker revealed significant treatment effects (p's<0.01) with significant dose-dependent effects (p<0.05).

### Behavioral analysis of DPC transplanted stroke rats

**Figure 5:**
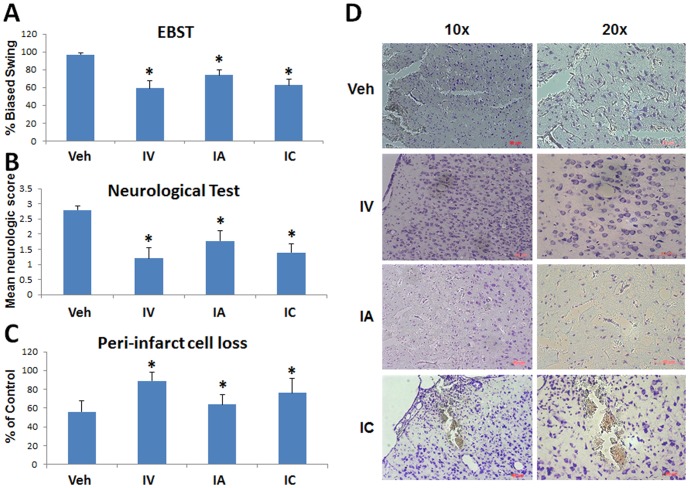
DPC grafts attenuate stroke behavioral and histological deficits. DPC transplanted stroke rats displayed significant improvement in their behavioral performance as revealed by decreased biased swing activity in EBST (A) and reduced impairment score in neurological test (B) compared to stroke rats that received vehicle only (*p<0.05). The transplanted stroke rats that received either IV or IC transplants exhibited significantly better behavioral recovery than those that received either IA-delivered DPCs or vehicle infusion. Additionally, transplantation of DPCs in stroke animals significantly reduced the peri-infarct cells loss in the striatum (C, D), again with much more robust rescue of the peri-infarct area in IV and IC transplanted stroke rats compared to IA-delivered DPCs or vehicle infusion (*p<0.05).

DPC transplanted stroke rats demonstrated significantly less impairment than the vehicle-infused stroke rats. EBST indicated decreased biased swing percent in transplanted stroke rats compared to those that received vehicle only (Figure 5A). ANOVA revealed significant treatment effects (F_3,38_ = 92.76, p<0.001) and pairwise comparisons between treatment groups were significant at p<0.05. Neurological testing revealed a reduction in impairment score in DPC transplanted stroke rats compared to those that received vehicle only (Figure 5B). ANOVA revealed significant treatment effects (F_3,38_ = 75.44, p<0.01) and pairwise comparisons between treatment groups were significant at p<0.05. In both tasks, all transplanted stroke animals were significantly improved than the vehicle only group, but the transplanted stroke rats that received either IV or IC transplants exhibited significantly better recovery than those that received the IA DPCs or vehicle infusion only.

### Histological analysis of DPC transplanted stroke rats

**Figure 6:**
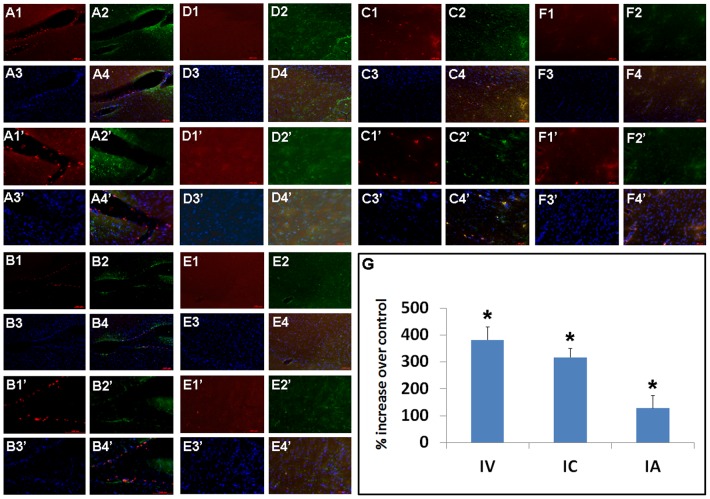
DPC grafts within the peri-infarct area co-localize with Hsp27 expression. The majority of grafted DPCs were deposited within the ipsilateral peri-infarct striatal site as detected by the fluorescent dye tracker PKH26. Less than 1% of the grafts survived with no detectable differences in graft persistence whether delivered IV, IC, or IA (p>0.05). Only sparse PKH26-labeled cells were found in the contralateral intact hemisphere across all three delivery routes. Significantly increased Hsp27 expression was juxtaposed to grafted DPCs and quantitative analyses of Hsp27 expression (G) in the peri-infarct striatal site revealed robust Hsp27 expression in transplanted brains compared to controls, more pronounced in IV- and IC-delivered DPCs compared to IA-administered DPCs (*p<0.05 vs. controls). A: IV-delivered cells, ipsilateral; B: IC-delivered cells, ipsilateral; C: IA-delivered cells, ipsilateral; D: IV-delivered cells, contralateral; E: IC-delivered cells, contralateral; F: IA-delivered cells, contralateral; 1: PKH26; 2: Hsp27; 3: DAPI; 4: Merged. 1–4: 10X; 1′–4′:20×.

Transplantation of DPCs in stroke animals significantly reduced the peri-infarct cells loss in the striatum (Figures 5C and 5D), which was detected in all cell delivery routes, but much more robust in IV and IC transplanted stroke rats, consistent with the results of a much better behavioral improvement with these two cell administration approaches. ANOVA revealed significant treatment effects (F_3,38_ = 59.81, p<0.0001) and pairwise comparisons between treatment groups were significant at p<0.05. Fluorescent microscopy revealed that the majority of grafted DPCs were deposited within the peri-infarct striatal site as revealed by dye tracking with PKH26 (Figure 6). Graft survival across the three cell delivery routes did not significantly differ, with graft persistence of 0.85%, 0.78%, 0.83% for IV, IA, and IC, respectively. Most of the grafted DPCs were found in the ipsilateral MCAo hemisphere, with only sporadic detection of PKH26-labeled cells in the contralateral intact hemisphere across all routes of cell delivery.

### Hsp27 as a potential mechanism of action for DPC-induced neuroprotection

**Figure 7:**
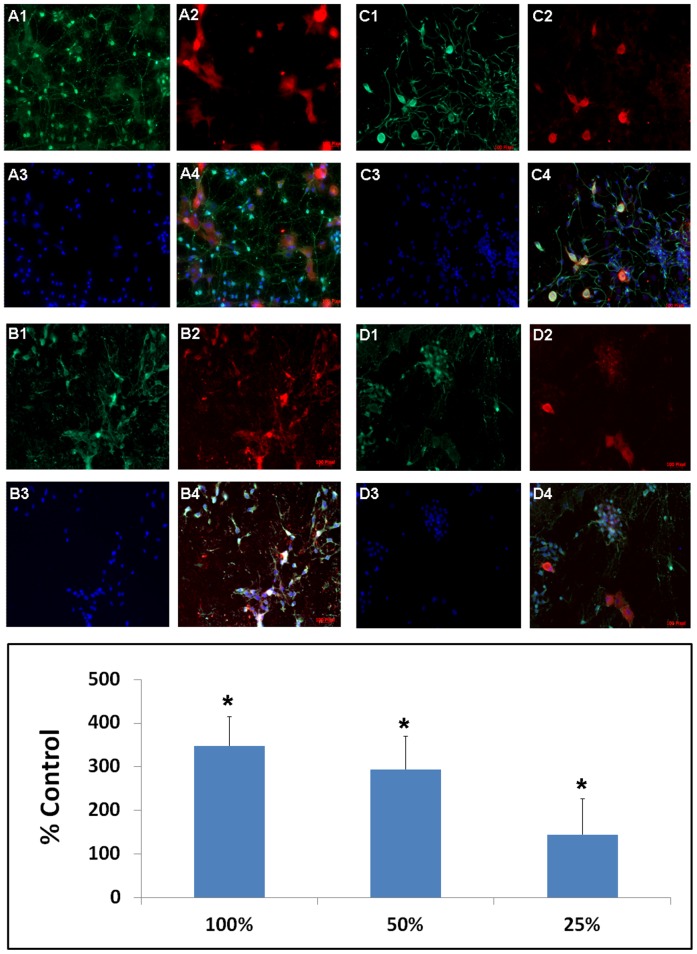
DPCs target Hsp27 for neuroprotection in vitro. Significantly elevated levels of Hsp27 expression were detected in OGD-exposed primary rat neural cells co-cultured with DPCs, which was most pronounced in GFAP-positive cells showing dose-dependent expression of Hsp27 (*p<0.05 vs. controls, i.e., 0%). A: 100% dose; B: 50% dose; C: 25% dose; D: 0% dose; 1: GFAP; 2: Hsp27; 3: DAPI; 4: Merged.

Stroke rats that received DPC transplants displayed significantly elevated Hsp27 expression localized around grafted DPCs near the peri-infarct striatal site (Figure 6). ANOVA revealed significant treatment effects (F_3,38_ = 56.15, p<0.001) and pairwise comparisons between treatments were significant (p<0.05). Interestingly, both IV and IC, which led to better behavioral and histological recovery, again showed much higher expression of Hsp27 compared to IA or controls (p<0.05). To further reveal the role of Hsp27 in DPC-induced neuroprotection, we also showed that primary rat neural cells co-cultured with DPCs and subjected to OGD exhibited a significantly higher level of Hsp27 expression in comparison to vehicle. In particular, significantly increased Hsp27 expression levels coincided with the upregulated GFAP expression in a dose-dependent fashion (Figure 7). ANOVA revealed significant treatment effects (F_3,9_ = 70.28, p<0.001) and pairwise comparisons between doses were significant (p<0.05).

## Discussion

The present study demonstrated that DPCs display stem cell-like properties and exert robust therapeutic benefits for ameliorating behavioral and histological deficits associated with experimental ischemic stroke models. We observed that co-cultured or transplanted DPCs protected against in vitro and in vivo models of stroke. Our findings highlighted the critical role of Hsp27 in DPC-mediated neuroprotection and warrants further investigations into Hsp-based therapeutics for abrogating stroke.

Immunocytochemical analysis of cultured DPCs revealed expression of markers for both stem and neural phenotypes, demonstrating both the stem cell properties of DPCs and their ability to differentiate into neural cells under appropriate cell culture conditions. Furthermore, our in vitro experiments revealed rat neural cells exposed to OGD conditions and co-cultured with DPCs exhibited dose-dependent improvement in cell survival and ATP production compared to the vehicle, indicating the importance of cell dose in achieving neuroprotection.

These in vitro observations of DPC–mediated neuroprotection were replicated in an animal model of stroke. Stroke rats that received DPC transplants exhibited significantly less behavioral deficits, observed by the improvements in motor and neurological impairments compared to vehicle. Histological data also reflected less ischemic cell loss in rats that received DPC transplants. Although the three cell routes of delivery employed in our study produced significant reductions in behavioral and histological deficits than vehicle-infused stroke animals, the IV and the IC routes generated more pronounced therapeutic outcomes than the IA cell administration. The dosage and timing optimal for each cell route, which may influence cell migration, deposition, and eventual functional effects of the grafts, will likely need to be determined in order to better understand the sub-optimal benefits seen in the IA route. Moreover, the therapeutic cell dose in vivo may need to consider the infarct size, in that the larger the infarction, the higher the cell dose may be required for achieving efficacy. However, although the 1∶1 in vitro ratio of DPCs to rat primary neural cells seemed optimal against OGD, DPCs may be operating under a paracrine/autocrine mechanism and targeting the anti-oxidative stress Hsp27 pathway in affording neuroprotection as we showed here. Accordingly, a threshold of cell dose may be required to achieve a therapeutic effect, but that the extent of neuroprotection may be carried out by therapeutic molecules, such as Hsp27, secreted by the transplanted DPCs.

Previous studies have implicated the role of Hsp27 as a neuroprotective element in ischemic brain injuries [43], [44], [45]. Our in vitro immunocytochemical analysis shows an increase in Hsp27 concentration in rat neural cells co-cultured with DPCs compared to that of the vehicle in response to OGD conditions. That the GFAP-positive glial cells appeared more co-localized with Hsp27 suggests that glial cell alterations seen in stroke are primarily reversed by DPC-induced neuroprotection. Similarly, our in vivo immunohistochemistry reveals increased concentration of Hsp27 localized around transplanted DPCs near the peri-infarct site. These results support Hsp27 as a key neuroprotective signaling pathway solicited by DPC transplantation, indicating that Hsp-based strategies may improve outcomes of cell therapy in stroke. Hsp in general has been implicated as an anti-oxidative stress protein that is upregulated after stroke [34], [35]. Moreover, Hsp is highly inducible in both glial and neuronal cells [36], [37] affording neuroprotective effects against ischemic insults [38], [39], thereby a molecule of interest that may be over-expressed in neural stem cells for therapeutic applications in stroke.

No specific treatment exists for dogs suffering from ischemic stroke but, our study demonstrates the neuroprotection afforded by DPCs both in vitro and in vivo in response to stroke conditions. These observations suggest that the transplantation of DPCs in dogs suffering from ischemic stroke may prove to be an effective treatment. It is worth mentioning that efforts into stem cell based regenerative medicine for animals are already underway as treatment for arthritis [46], [47], tendon repair [48], and certain autoimmune disorders [49], [50], [51]. Vet-Stem, a company for regenerative veterinary medicine, has used these findings to justify the use of stem cell treatments in various animals. Vet-Stem recently demonstrated the efficacy of autologous transplant of adipose derived mesenchymal stem cells (AD-MSCs) as a treatment for chronic osteoarthritis in dogs [52], [53]. Unfortunately, autologous transplantation poses as a technically challenging approach in the clinic, and may limit the target patient population. Alternatively, xenografts (for animals), as well as allografts with HLA matching, may be equally potential transplantation strategies for placenta-derived stem cells. Indeed, on-going clinical trials for stroke have pursued allograft transplantation of stem cells [25].

Our results are consistent with other recent preclinical studies of placenta transplantation stroke therapy. Rats subjected to a two-hour MCAo and subsequently treated with human placenta-derived mesenchymal-like stem cells exhibited significantly improved recovery compared to vehicle [20]. Similarly, rats given permanent MCAo and then treated with human maternal placenta-derived mesenchymal stromal cells demonstrated improved recovery compared to control [54]. In concert with these studies, our study shows that the dog placenta is a viable source of stem cells and shows the therapeutic benefit of DPC transplantation in response to ischemic injury. These results highlight the possible benefits of investigation into the efficacy of autologous transplant of placenta cells in dog stroke patients, especially when considering the lack of available treatment for ischemic injury in dogs.

In line with the envisioned clinical product of stem cell transplantation in stroke patients, large animals, including dogs as in the present study, have been investigated as possible models for human stroke therapy. Such large animal modeling has also been pursued in pigs [55]. A source of stem cells has been identified in the subventricular zone of piglets, which displayed robust ability to form neurospheres and high capacity to differentiate into neurons, astrocytes, and oligodendrocytes. Altogether these results provide a mechanism-based evidence for the observed functional effects of stem cells, building a strong case for pre-clinical trials in large animal models for translational route of lab-to-clinic application of cell therapy for stroke [56].

Veterinary and clinical applications of placenta-derived MSC therapy may require cost effective technologies to produce these cells in a large industrial scale may be a challenge [55], [56]. For animal applications, veterinary uses of stem cell therapy have already emerged, with veterinary grade AD-MSC transplant therapy commercially available since 2003 [53]. Assessment of therapeutic benefits of our DPCs for treatment of stroke in dogs will be the short-term goal. For human applications, technical challenges associated with collection and storage of autologous human placenta stem cells, similar to umbilical cord blood banks, will need to be addressed in parallel with preclinical testing of safety and efficacy of placenta-derived MSCs.

**Figure 8:**
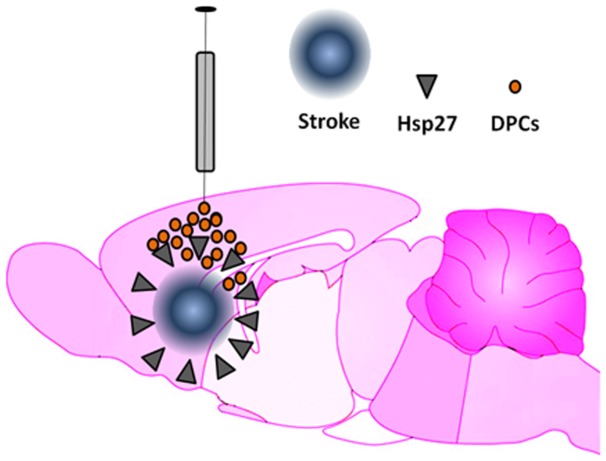
Hsp-27-mediated neuroprotection following DPC transplantation in stroke. Hsp27 appears to be targeted by DPCs for neuroprotection in stroke. Following intracerebral transplantation, DPCs survived in the stroke brain and elevate Hsp27 expression which may afford rescue of the ischemic penumbra proximal, as well as distal from the transplant site. Although the transplanted cells do not migrate far from the transplant site, the anti-oxidative stress protein Hsp27 is able to amplify the neuroprotection.

Our findings provide evidence of the therapeutic efficacy of placenta cells for ischemic stroke. The cell route of delivery is critical to the functional outcome. Targeting the Hsp27 signaling pathway may improve the therapeutic benefit (Figure 8). Long-term studies on safety and efficacy are warranted to allow a systematic assessment of clinical applications of autologous human placenta cell transplantation in stroke patients.
